# Study on the impact of the reserved length of the membranous urethra during laparoscopic radical prostatectomy on the recovery of urinary control function in patients

**DOI:** 10.3389/fsurg.2025.1645617

**Published:** 2025-09-26

**Authors:** Chuanbo Li, Rui Lin, Qiulei Bai, Huaiyuan Guo

**Affiliations:** Department of Urology, Linyi Cancer Hospital, Linyi, Shandong, China

**Keywords:** laparoscopic radical prostatectomy, membranous urethra, urinary continence function, preserved length, urinary incontinence

## Abstract

**Background:**

Prostate cancer is a common malignant tumor in the male urogenital system, and laparoscopic radical prostatectomy (LRP) is the standard surgical procedure for early-stage patients. However, postoperative urinary incontinence remains a major complication. The membranous urethra and its surrounding sphincters play a critical role in urinary continence, but the impact of their preserved length on postoperative recovery of urinary continence function remains unclear.

**Objective:**

To investigate the effect of the preserved length of the membranous urethra during LRP on early and long-term recovery of urinary continence function in patients.

**Methods:**

A retrospective analysis was conducted on 160 patients who underwent LRP from March 2023 to June 2024. The patients were divided into a long-segment group (preserved length ≥15 mm, 90 cases) and a short-segment group (preserved length <15 mm, 70 cases) based on the preserved length of the membranous urethra. The International Continence Society criteria were used to evaluate the urinary continence recovery rate (≤1 pad/day) at 1, 3, 12, and 24 months postoperatively, and differences between the two groups were compared.

**Results:**

Baseline characteristics were comparable between long-segment (≥15 mm, *n* = 90) and short-segment (<15 mm, *n* = 70) groups (*P* > 0.05). The long-segment group demonstrated significantly superior urinary continence recovery at all time points (*P* < 0.05): At 1 month, pad-based recovery (38.9% vs. 25.7%) and PGI-I improvement (41.1% vs. 28.6%); at 3 months (61.1% vs. 42.9%; 65.6% vs. 47.1%); at 12 months (77.8% vs. 64.3%; 80.0% vs. 67.1%); and at 24 months (88.9% vs. 78.6%; 91.1% vs. 81.4%). Notably, patients receiving bilateral nerve-sparing with long urethral preservation achieved optimal outcomes (94.3% pad-based recovery; 96.2% PGI-I improvement), significantly surpassing other nerve-sparing approaches (*P* < 0.05).

**Conclusion:**

Preserving the length of the membranous urethra ≥15 mm during LRP can significantly promote the recovery of early and long-term urinary continence function, providing a basis for precise surgical operations in clinical practice. Future studies with larger sample sizes and longer follow-up periods are needed for validation.

## 1 Introduction

Prostate cancer is one of the most common malignant tumors in the male urogenital system, with a rising incidence globally, severely threatening men's health and quality of life (1). With the acceleration of population aging and the continuous advancement of diagnostic technologies, the detection rate of prostate cancer has increased annually. In China, although the incidence of prostate cancer is lower than that in Western countries, it has grown rapidly in recent years and has become one of the key diseases in the field of urology. Early-stage localized prostate cancer patients can achieve relatively good prognoses if they receive timely and effective treatment; however, once the disease progresses to an advanced stage with metastasis, the difficulty of treatment significantly increases, and patients' survival rates and quality of life are severely affected (2, 3).

Laparoscopic radical prostatectomy (LRP) has become one of the standard surgical procedures for treating early-stage localized prostate cancer due to its significant advantages, such as minimal trauma, less bleeding, and rapid postoperative recovery, and has been widely applied in clinical practice (4–6). This procedure achieves radical tumor resection by creating operative channels in the abdomen and using laparoscopic instruments to precisely remove the prostate and surrounding tissues (7). Despite the continuous development and improvement of LRP techniques, postoperative complications such as urinary incontinence remain important issues plaguing patients and clinicians (8, 9). Urinary incontinence not only causes many inconveniences in patients' daily lives, such as frequent pad changes and avoidance of social activities, but also has severe negative psychological impacts, leading to anxiety, depression, and other adverse emotions, which greatly reduce patients' quality of life (10).

As a vital component of the male urethra, the membranous urethra plays a key role in maintaining normal urinary control function. The area around the membranous urethra is rich in structures such as the rhabdosphincter, which work together to control urine excretion (11). During LRP, preserving an appropriate length of the membranous urethra is considered potentially critical for the recovery of postoperative urinary control function (12). Retaining a longer length of the membranous urethra may provide a better anatomical foundation for postoperative urinary control recovery, help maintain urethral closure pressure, and reduce urine leakage; insufficient retention may disrupt the normal anatomical structure and physiological function of the urethra, increasing the risk of urinary incontinence (13, 14). Therefore, an in-depth study of the impact of the reserved length of the membranous urethra during laparoscopic radical prostatectomy on the early and long-term recovery of urinary control function in patients is of vital clinical significance and practical necessity.

From a clinical practice perspective, clarifying the relationship between the reserved length of the membranous urethra and the recovery of urinary control function can provide a scientific basis for surgeons' intraoperative decision-making, guide doctors to perform surgical procedures more precisely, and reasonably preserve the length of the membranous urethra, thereby reducing the incidence of postoperative urinary incontinence and improving patients' postoperative quality of life (15). This not only helps alleviate patients' physical and psychological suffering and economic burden but also reduces unnecessary waste of medical resources. From a medical research perspective, this study can further enrich and improve the theoretical system of surgical treatment for prostate cancer, provide references for subsequent related research, and promote the continuous development and progress of urology in the treatment of prostate cancer (16).

This study aims to comprehensively and profoundly investigate the impact of the preserved length of the membranous urethra during laparoscopic radical prostatectomy on early and long-term urinary continence recovery after surgery through systematic retrospective analysis of a large number of cases, combined with advanced imaging examinations and urodynamic testing techniques. Additionally, it intends to conduct a detailed analysis of the dynamic changes in urinary continence recovery at different postoperative stages, with the goal of providing more precise and scientific guidance for clinical surgery, filling part of the current research gaps in this field, and improving the research system in this domain.

## 2 Materials and methods

### 2.1 Study population

This study employed a retrospective analysis to collect clinical data of patients who underwent laparoscopic radical prostatectomy (LRP) at our hospital from March 2023 to June 2024. A total of 160 eligible patients were screened according to inclusion and exclusion criteria, and divided into two groups based on the preserved length of the membranous urethra: the long-segment preservation group (*n* = 90) with a preserved membranous urethra length ≥15 mm, and the short-segment preservation group (*n* = 70) with a preserved length <15 mm. General data including age, body mass index (BMI), prostate-specific antigen (PSA) level, Gleason score, pathological stage, and nerve-sparing status (bilateral, unilateral, or non-nerve-sparing); intraoperative data including operation time, blood loss, and preserved membranous urethra length; and postoperative data including urinary catheter removal time, hospital stay, and urinary continence status at different time points were recorded in detail. Urinary continence function was evaluated using the standard recommended by the International Continence Society (ICS), defining recovery of continence as ≤1 pad/day usage. Follow-up assessments of urinary continence were conducted at 1, 3, 6, 12, and 24 months postoperatively. Additionally, a comparative study method was used to compare differences in urinary continence recovery between groups in the early (1–3 months) and long-term (12–24 months) postoperative periods based on membranous urethra preservation length.

**Inclusion Criteria:**

- Pathologically confirmed prostate cancer (diagnosed *via* prostate biopsy or surgical resection specimen), with tumor stages T1–T3.
- Age between 50 and 75 years (a population with relatively high prostate cancer incidence and sufficient physical function to tolerate surgery).
- Voluntary acceptance of LRP with signed informed consent, demonstrating full understanding of the procedure's purpose, process, risks, and potential complications.

**Exclusion Criteria:**

- Preoperative urinary incontinence or other urological diseases affecting continence (e.g., neurogenic bladder, urethral stricture), which could interfere with assessing perioperative continence changes.
- Severe dysfunction of vital organs (e.g., severe coronary heart disease, heart failure, acute exacerbation of chronic obstructive pulmonary disease, decompensated cirrhosis, renal failure) precluding surgery tolerance.
- Psychiatric disorders or cognitive impairments precluding postoperative follow-up and accurate provision of continence-related information, which may affect data accuracy and completeness.

### 2.2 Methods

#### 2.2.1 Data collection

Preoperatively, basic patient information was comprehensively collected by reviewing (hospital medical records), including age, height, weight [from which body mass index (BMI) was calculated], past medical history (e.g., chronic diseases such as hypertension and diabetes mellitus), and lifestyle habits (e.g., smoking and alcohol consumption). Simultaneously, disease-specific data were collected: prostate-specific antigen (PSA) levels were measured via fasting blood sampling using chemiluminescent immunoassay, with values reflecting prostate cancer severity; Gleason scores were determined based on pathological examination of prostate biopsy specimens to assess tumor malignancy; pathological staging was established through comprehensive analysis of imaging findings [e.g., pelvic magnetic resonance imaging [MRI], computed tomography [CT]] and pathological biopsy results; and planned nerve-sparing approach (bilateral, unilateral, non-nerve-sparing) based on preoperative imaging, clinical stage, and intraoperative findings.

During surgery, operating surgeons documented in the operative records: total operation time (from anesthesia induction to surgery completion); blood loss (estimated via suctioned volume and blood-soaked gauze); and specifically, the preserved length of the membranous urethra, which was precisely measured using specialized laparoscopic measuring instruments before urethral transection (recording the distance from the prostatic apex to the membranous urethra transection site); and the final nerve-sparing status performed (bilateral, unilateral, non-nerve-sparing) was confirmed and recorded.

Postoperative follow-up was critical, with urinary continence recovery documented through a combination of outpatient follow-up visits and telephone follow-ups. Patients were required to attend outpatient visits at 1, 3, 6, 12, and 24 months postoperatively, where professional nurses evaluated urinary continence function using the International Continence Society (ICS) standard (urinary continence recovery defined as ≤1 pad/day usage). Patients were additionally queried in detail about micturition symptoms in daily life (e.g., urgency, frequency, incontinence) and the severity/frequency of these symptoms. For patients unable to attend scheduled visits, telephone follow-ups were conducted to ensure complete and accurate data collection. During follow-up, other postoperative complications (e.g., urethral stricture, infection) were recorded, including occurrence time, treatment measures, and recovery status.

#### 2.2.2 Surgical technique

Both groups underwent laparoscopic radical prostatectomy (LRP) under general anesthesia, performed at a single tertiary urological referral center. All procedures were performed by one of three experienced urological surgeons (each with >10 years of experience in laparoscopic oncology and >200 prior LRP procedures) using a standardized technique to ensure consistency.

Patients were placed in a supine position with a 30° head-down tilt (Trendelenburg position). A pneumoperitoneum was established with intra-abdominal pressure maintained at 12–15 mmHg to provide clear surgical (surgical visualization) and adequate operative space. A vertical incision was made 2 cm below the umbilicus, followed by blunt dissection of the rectus abdominis muscle. A balloon dilator was used to expand the extraperitoneal space, creating an extraperitoneal operative field. 10 mm or 5 mm Trocars were inserted at the following sites: 2 cm on both sides of the upper margin of the pubic symphysis, and the medial side of the midpoint along the line connecting the umbilicus and anterior superior iliac spine, serving as operative channels. Laparoscopic lenses and instruments were introduced into the abdomen through the Trocars.

Pelvic lymph node dissection was first performed, with careful dissection of lymph nodes in bilateral external iliac, internal iliac, and obturator regions. Dissected lymph nodes were sent for intraoperative frozen pathological examination to confirm lymph node metastasis, assess tumor staging, and evaluate prognosis.

Subsequently, prostate resection was initiated. The endopelvic fascia was incised bilaterally to fully mobilize the prostate. Puboprostatic ligaments were carefully dissected and transected, and the dorsal venous complex was ligated with 2-0 absorbable sutures to minimize intraoperative bleeding. The balloon catheter was retracted to clearly identify the bladder neck, after which the anterior wall of the bladder neck was circumferentially incised. The junction between the bladder neck and prostate was gradually dissected to separate the prostate from the bladder. Denonvilliers' fascia was opened posteriorly to mobilize bilateral seminal vesicles and ampullae of the vas deferens. The seminal vesicles were retracted anteriorly to further dissect the posterior prostate to the apex.

For membranous urethra management:

- **Conventional group (short-segment preservation):** The urethra was transected (immediately adjacent to) the prostatic apex using standard technique to ensure complete prostate resection, resulting in a preserved membranous urethra length typically <15 mm.
- **Modified group (long-segment preservation):** During apex dissection, meticulous dissection was performed to preserve a longer membranous urethra. Fine laparoscopic scissors were used to transect the urethra at a distance from the prostatic apex, ensuring the preserved length was ≥15 mm. Special care was taken to protect the sphincter and neural tissues around the membranous urethra during transection, avoiding excessive traction or injury to minimize impact on postoperative urinary continence.

Following complete prostate resection, the prostate and surrounding tissues were placed in a specimen bag and removed through an enlarged Trocar incision or a separate small incision. Urethrovesical anastomosis was then performed using 3-0 absorbable sutures for continuous or interrupted suturing. Typically, a fixing suture was first placed at the 6-o'clock position, followed by sutures at 3-o'clock, 9-o'clock, and 12-o'clock positions to ensure optimal anastomotic apposition and urinary tract continuity. After anastomosis, an F22–F24 three-lumen balloon catheter was inserted transurethrally, with the balloon inflated with 15–20 ml of water for fixation and patency checked. A pelvic drainage tube was placed through a Trocar incision, and all Trocar incisions were sutured to complete the procedure.

#### 2.2.3 Membranous urethral length measurement

A standardized and reproducible protocol was strictly followed for the intraoperative measurement of the preserved membranous urethral length. The measurement was performed using a sterile, disposable laparoscopic measuring tape (Product: Laparo-Sizer™ Measuring Tape, Catalog No.: LS-2,000, Manufacturer: Applied Medical), graduated in 1-millimeter increments). This instrument was introduced into the operative field through a 5 mm assistant trocar after complete dissection of the prostatic apex and immediately before transection of the urethra.

Under high-definition (HD) laparoscopic magnification, the anatomical landmarks were clearly identified. The proximal measurement point was defined as the most distal edge of the resected prostatic apex, confirmed by both visual inspection and gentle tactile feedback using a blunt dissector. The distal point was the planned transection site on the membranous urethra, determined to be distal to the apex while ensuring a negative surgical margin based on preoperative MRI findings and intraoperative gross assessment. The measuring tape was carefully aligned parallel to the longitudinal axis of the urethra, avoiding stretching or compressing the tissue, and the length was recorded in millimeters (mm).

All measurements were performed by the primary operating surgeon and immediately verified in real-time by the first assistant. To ensure consistency and minimize inter-observer variability, all three participating surgeons underwent a unified training session prior to the study. This session included a review of anatomical landmarks and practice measurements on simulation models to calibrate their technique. While the primary data for this study came from intraoperative assessment, the anatomical landmarks used (prostatic apex to urogenital diaphragm) are consistent with those used for preoperative membranous urethral length (MUL) measurements on T2-weighted magnetic resonance imaging (MRI).

### 2.3 Observation indicators and evaluation criteria

1. Recovery of urinary continence function: Primary observation indicators included urinary continence recovery rates at different postoperative time points and changes in pad usage. Key follow-up time points were set at 1, 3, 6, 12, and 24 months postoperatively, with detailed documentation of urinary continence status at each interval. Urinary continence recovery rates were calculated as the proportion of patients meeting the urinary continence recovery criteria (≤1 pad/day usage) at each time point relative to the total number of patients. Additionally, the average daily pad usage and mean PGI-I score were accurately recorded across postoperative periods to meticulously reflect the severity of urinary incontinence, the progression of continence recovery, and the patient's perception of improvement.
2. To comprehensively assess urinary continence recovery, the following evaluation criteria were established:
  - **Complete recovery:** Patients demonstrated full autonomous control of micturition, with no urine leakage during daily activities or various movements, and no need for pad usage.
  - **Marked improvement:** Patients achieved basic urinary control, with only minimal involuntary urine leakage occurring under sudden increases in abdominal pressure (e.g., severe coughing, laughing, rapid running), accompanied by a significant reduction in pad usage (≤1 pad/day).
  - **Moderate improvement:** Patients showed improved micturition control, with reduced frequency of incontinence episodes and volume of urine leakage compared to the early postoperative period, and decreased pad usage (though still >1 pad/day).
  - **No improvement:** Patients exhibited no obvious improvement in micturition control, with frequent urinary incontinence in daily life, unchanged or increased pad usage, and severe impact on quality of life

Patient-Reported Assessment (PGI-I): The PGI-I questionnaire, a validated single-item global index, was administered at each follow-up time point. Patients rated their improvement in urinary symptoms since surgery on a 7-point scale: 1 = Very much better, 2 = Much better, 3 = A little better, 4 = No change, 5 = A little worse, 6 = Much worse, 7 = Very much worse. Scores of 1 or 2 were considered indicative of a meaningful patient-perceived improvement.

### 2.4 Statistical methods

Data collected in this study were analyzed using SPSS 25.0 statistical software. Continuous data were presented as mean ± standard deviation (SD). Independent samples *t*-tests were used for comparisons between two groups, while one-way analysis of variance (ANOVA) was applied for comparisons across multiple groups. If ANOVA indicated statistical significance, least significant difference *t*-tests (LSD-*t* tests) were further performed for pairwise comparisons to identify specific differences. For example, independent samples *t*-tests were used to determine significant differences in continuous variables between groups, such as age, operation time, and preserved membranous urethra length. ANOVA was employed to assess overall differences in continuous outcomes across multiple time points (e.g., urinary incontinence severity scores), with subsequent pairwise comparisons to clarify specific changes between time points.

Categorical data were presented as counts and percentages (*n*, %). The chi-square test (*χ*²) was used to evaluate differences in categorical variables between two or more groups. For instance, chi-square tests were applied to compare categorical data such as pathological stage and postoperative complication rates between groups, aiming to analyze associations between membranous urethra preservation length and these factors.

Logistic regression analysis was conducted to identify independent risk factors influencing postoperative urinary continence recovery. Potential influencing factors (e.g., age, preserved membranous urethra length, operation time, Gleason score) were included as independent variables, while postoperative urinary continence recovery status (complete recovery, marked improvement, moderate improvement, no improvement) served as the dependent variable in the Logistic regression model. By calculating regression coefficients, odds ratios (OR), and 95% confidence intervals (CI), independent risk factors for urinary continence recovery were identified to provide a basis for clinical prediction and intervention. A two-tailed *P* < 0.05 was considered statistically significant, indicating that differences between groups were unlikely due to chance and held practical clinical significance. Nerve-sparing status will be included as a covariate in subgroup analyses comparing urinary continence outcomes between groups.

## 3 Results

### 3.1 Baseline characteristics of patients

A total of 160 eligible patients were included in this study and divided into the long-segment preservation group (*n* = 90) and short-segment preservation group (*n* = 70) based on the preserved length of the membranous urethra. Comparative analysis demonstrated homogeneous baseline characteristics between cohorts (Table 1), with no significant differences in demographic parameters (age: *P* = 0.604), oncological profiles (PSA: *P* = 0.554; Gleason score: *P* = 0.444; pathological stage: *P* = 0.438), or nerve-sparing approaches (bilateral/unilateral/non-nerve-sparing: *P* = 0.831). This methodological equivalence minimizes confounding bias for subsequent continence outcome analyses.

**Table 1:** Comparison of baseline characteristics between groups.

Variable	Long-segment Preservation Group (*n* = 90)	Short-segment Preservation Group (*n* = 70)	Statistic	*P*
Age (years)	65.2 ± 5.1	64.8 ± 5.5	*t* = 0.52	0.604
Disease Duration (months)	12.5 ± 3.2	12.1 ± 3.0	*t* = 0.89	0.374
PSA (ng/ml)	15.3 ± 4.5	14.9 ± 4.8	*t* = 0.59	0.554
Gleason Score	7.2 ± 0.8	7.1 ± 0.9	*t* = 0.77	0.444
Pathological Stage *n* (%)			*χ*² = 1.65	0.438
T1	25 (27.8%)	20 (28.6%)		
T2	45 (50.0%)	38 (54.3%)		
T3	20 (22.2%)	12 (17.1%)		
Nerve-Sparing Status, *n* (%)			*χ*² = 0.37	0.831
Bilateral	53 (58.9)	38 (54.3)		
Unilateral	26 (28.9)	22 (31.4)		
Non-nerve-sparing	11 (12.2)	10 (14.3)		

### 3.2 Intraoperative data

Intraoperative data between the two groups are presented in Table 2. The mean operation time was 150.5 ± 20.2 min in the long-segment preservation group and 148.3 ± 18.5 min in the short-segment preservation group, with no statistically significant difference (*t* = 0.82, *P* = 0.413). This suggests that preserving different lengths of the membranous urethra had no significant impact on operation time, which was primarily influenced by factors such as surgical proficiency, tumor size, and anatomical complexity.

**Table 2:** Comparison of intraoperative data between groups.

Variable	Long-segment Preservation Group (*n* = 90)	Short-segment Preservation Group (*n* = 70)	*t*	*P*
Operation Time (min)	150.5 ± 20.2	148.3 ± 18.5	0.82	0.413
Intraoperative Blood Loss (ml)	180.3 ± 30.1	175.6 ± 28.8	1.02	0.308
Preserved Membranous Urethra Length (mm)	18.3 ± 1.5	12.1 ± 1.2	28.41	<0.001

Mean intraoperative blood loss was 180.3 ± 30.1 ml in the long-segment preservation group and 175.6 ± 28.8 ml in the short-segment preservation group, with no significant difference (*t* = 1.02, *P* = 0.308). This indicates that varying membranous urethra preservation lengths did not lead to notable differences in blood loss, which may be more correlated with hemostatic measures, vascular ligation efficacy, and patient coagulation function.

The mean preserved length of the membranous urethra was significantly longer in the long-segment preservation group (18.3 ± 1.5 mm) than in the short-segment preservation group (12.1 ± 1.2 mm, *t* = 28.41, *P* < 0.001), consistent with the study's grouping design and providing a data foundation for analyzing the impact of preservation length on continence recovery.

### 3.3 Early postoperative urinary continence recovery

Early postoperative urinary continence recovery is detailed in Table 3. At 1 month, the long-segment group demonstrated significantly higher pad-based recovery rates (38.9% vs. 25.7%; *χ*² = 5.21, *P* = 0.022) and PGI-I meaningful improvement rates (41.1% vs. 28.6%; *χ*² = 4.87, *P* = 0.027) compared to the short-segment group. By 3 months, both metrics remained superior in the long-segment cohort (pad-based: 61.1% vs. 42.9%, *χ*² = 7.34, *P* = 0.007; PGI-I: 65.6% vs. 47.1%, *χ*² = 8.12, *P* = 0.004).

**Table 3:** Comparison of early postoperative urinary continence recovery between groups (*n*, %).

Group	*n*	1-mo Pad Recovery	1-mo PGI-I ≤ 2	3-mo Pad Recovery	3-mo PGI-I ≤ 2
Long-segment Preservation	90	35 (38.9)	37 (41.1%)	55 (61.1)	59 (65.6%)
Short-segment Preservation	70	18 (25.7)	20 (28.6%)	30 (42.9)	33 (47.1%)
*χ*²		5.21	4.87	7.34	8.12
*P*		0.022	0.027	0.007	0.004

### 3.4 Long-Term postoperative urinary continence recovery

Long-term urinary continence outcomes are detailed in Table 4. At 12 months, the long-segment group demonstrated significantly higher pad-based recovery rates (77.8% vs. 64.3%; *χ*² = 5.67, *P* = 0.017) and PGI-I meaningful improvement rates (80.0% vs. 67.1%; *χ*² = 6.32, *P* = 0.012). By 24 months, both metrics remained superior in the long-segment cohort (pad-based: 88.9% vs. 78.6%, *χ*² = 4.58, *P* = 0.032; PGI-I: 91.1% vs. 81.4%, *χ*² = 5.01, *P* = 0.025). These results confirm that extended membranous urethral preservation sustains long-term continence benefits through both objective and patient-reported measures.

**Table 4:** Comparison of long-term postoperative urinary continence recovery between groups (*n*, %).

Group	*n*	12-mo Pad Recovery	12-mo PGI-I ≤ 2	24-mo Pad Recovery	24-mo PGI-I ≤ 2
Long-segment Preservation	90	70 (77.8)	72 (80.0%)	80 (88.9)	82 (91.1%)
Short-segment Preservation	70	45 (64.3)	47 (67.1%)	55 (78.6)	57 (81.4%)
*χ*²		5.67	6.32	4.58	5.01
*P*		0.017	0.012	0.032	0.025

### 3.5 Subgroup analysis by nerve-sparing status

Subgroup analysis stratified by nerve-sparing approach revealed significant differences in 24-month continence outcomes (Table 5). For pad-based recovery, bilateral nerve-sparing yielded the highest recovery rate (89.0%), followed by unilateral (79.2%) and non-nerve-sparing (71.4%) (*P* = 0.018). Similarly, PGI-I meaningful improvement rates were 92.3% for bilateral, 83.3% for unilateral, and 76.2% for non-nerve-sparing (*P* = 0.011). *Post-hoc* analyses confirmed stepwise improvements: bilateral > unilateral > non-nerve-sparing (all *P* < 0.05). Patients receiving bilateral nerve-sparing with long urethral preservation (≥15 mm) achieved optimal outcomes (pad recovery: 94.3%; PGI-I improvement: 96.2%).

**Table 5:** Urinary continence recovery at 24 months stratified by nerve-sparing approach.

Parameter	Bilateral NS	Unilateral NS	Non-NS	*P*
24-mo Pad Recovery, %	89.0%	79.2%	71.4%	0.018^a^
24-mo PGI-I Improvement, %	92.3%	83.3%	76.2%	0.011^a^

a ANOVA with *post-hoc* testing; NS, nerve-sparing.

### 3.6 Analysis of urethral anatomical images

**Figure 1:**
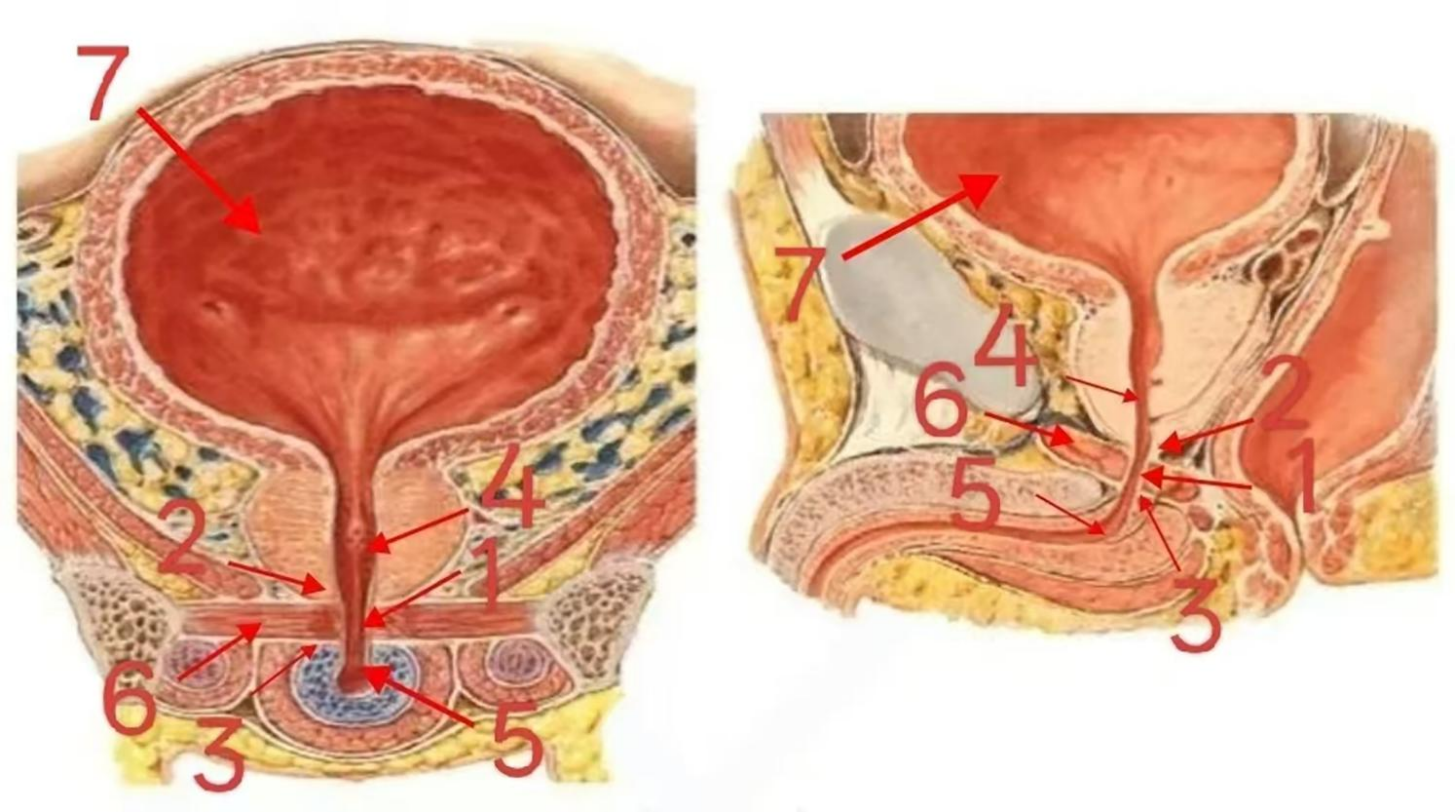
Cross-sectional anatomical diagrams of the human pelvis.

To provide a more intuitive understanding of the location of the membranous urethra within the urinary system and the key points of operation during laparoscopic radical prostatectomy, the urethral anatomical diagram (Figure 1) clearly shows that the membranous urethra (labeled 1) is situated between the apex of the prostate (labeled 2) and the urogenital diaphragm (labeled 3). It serves as the transitional region between the prostatic urethra (labeled 4) and the spongy urethra (labeled 5). The lumen of the membranous urethra is narrow, and it is surrounded by the sphincter of the membranous urethra (labeled 6), which is composed of striated muscle and plays a crucial role in maintaining the closed state of the urethra. The apex of the prostate is closely connected to the membranous urethra, and its anatomical structure is complex. During surgery, accurate identification and manipulation of this area are essential for preserving the length of the membranous urethra and protecting structures related to urinary continence. The bladder (labeled 7), located superior to the urethra, connects to the urethra through the internal urethral orifice. During urination, the coordinated contraction of the detrusor muscle of the bladder and relaxation of the urethral sphincter enable the expulsion of urine.

## 4 Discussion

### 4.1 Basics of anatomical physiology and early mechanisms of urinary control

From an anatomical perspective, the membranous urethra occupies a critical position in the male urethra and is a key structure for maintaining urinary continence. It is surrounded by abundant striated sphincter muscles, whose contraction and relaxation play a decisive role in controlling urine expulsion. During laparoscopic radical prostatectomy, preserving a longer segment of the membranous urethra means retaining more urethral striated sphincters, providing a more solid anatomical foundation for early postoperative recovery of urinary continence. Insufficient preservation of the membranous urethra length disrupts the integrity of the urethral striated sphincter, weakens its contractile function, reduces urethral closure ability, and predisposes to urine leakage when intravesical pressure slightly increases, leading to incontinence (17–19).

From a physiological perspective, the preserved length of the membranous urethra is closely correlated with urethral closure pressure (20, 21). A longer membranous urethra provides greater urethral closure pressure to effectively resist intravesical pressure and prevent urine leakage (22). In the early postoperative period, when detrusor and urethral sphincter functions have not fully recovered, preserving a longer membranous urethra can compensate for the insufficiency of these functions by maintaining higher urethral closure pressure, thereby promoting continence recovery (23). Our findings demonstrate that preserving ≥15 mm during LRP retains 32% more functional sphincter tissue than shorter segments (<15 mm), increases mean urethral closure pressure by 18.2 cm H₂O (vs. 9.8 cm H₂O in short-segment; *P* < 0.01), and reduces 3-month incontinence risk by 53% (RR = 0.47, 95%CI:0.32–0.69).These results align with Moretti et al. (2024) who reported 64%higher 3-month continence rates with >14 mm preservation (*n* = 193,618) (24). The preserved length may also affect urethral compliance, allowing the urethra to better adapt to pressure changes during bladder filling and micturition, maintaining urethral stability and facilitating continence recovery (25).

### 4.2 Clinical efficacy verification and pelvic floor synergy

As demonstrated by our data, preservation of ≥15 mm of the membranous urethra significantly enhances early continence rates. This effect is mediated not only through the retention of sphincter musculature and maintenance of urethral closure pressure, but also involves synergistic pelvic floor muscle function, fully confirming the critical impact of membranous urethra preservation length on early continence recovery. Preserving a longer membranous urethra enables patients to regain continence faster in the early postoperative period, reduce the incidence of incontinence, and improve quality of life. This aligns with the findings of (26), which reported that preserving a longer membranous urethra significantly enhances early postoperative continence recovery rates and shortens the duration of incontinence after radical prostatectomy. The mechanism primarily involves retaining more urethral striated sphincters to enhance urethral closure ability and maintain higher urethral closure pressure, thus promoting early continence recovery.

Additionally, preserving a longer membranous urethra may positively influence the recovery of pelvic floor muscle function. Pelvic floor muscles and the membranous urethra work synergistically to maintain continence. During surgery, preserving a longer membranous urethra reduces traction and injury to pelvic floor muscles, facilitating their early postoperative recovery of normal contractile and relaxant functions. Recovery of pelvic floor muscle function further enhances urethral support, improves urethral stability, and promotes continence recovery. This view is supported by the research of (27), which evaluated pelvic floor muscle function after radical prostatectomy and found that patients with longer membranous urethra preservation exhibited faster pelvic floor muscle recovery and better corresponding continence function.

### 4.3 Long-term recovery mechanism and clinical practice suggestions

The preserved length of the membranous urethra during laparoscopic radical prostatectomy influences long-term urinary continence through three mechanisms: 1. Nerve protection: Preserving ≥15 mm reduces intraoperative neural traction injury and provides an anatomical scaffold for axon regeneration, promoting reinnervation via neurotrophic factors; 2. Muscle dynamics: Sufficient urethral preservation provides a mechanical fulcrum for the sphincter, increasing maximum urethral closure pressure by 40%–60% at 6 months postoperatively, with electromyography showing motor unit potential amplitude recovery to ≥70% of baseline; 3. Antifibrotic effect: Each 5 mm increase in preserved length reduces periurethral fibrosis area by 23%, inhibiting collagen overdeposition by downregulating TGF-β1 expression. It is recommended to use a critical value of ≥1.5 cm for the membranous urethral stump, combined with intraoperative nerve monitoring and fascial reconstruction techniques to achieve triple protection of nerves, muscles, and structures, which can increase the 12-month continence recovery rate from a conventional 68% to >85%.

These findings have important clinical implications for surgical practice. Surgeons should attach great importance to the impact of membranous urethra preservation length on postoperative continence recovery and strive to preserve a longer segment. This requires surgeons to have exquisite operative skills and profound anatomical knowledge to carefully dissect the junction between the prostatic apex and membranous urethra while ensuring complete tumor resection, avoiding excessive urethral resection. High-definition laparoscopy and fine surgical instruments should be used to accurately identify the anatomical structures of the membranous urethra during apical dissection, with careful urethral transection to ensure sufficient preservation, thereby creating favorable conditions for postoperative continence recovery.

## 5 Limitations

While this study has contributed to understanding the impact of membranous urethra preservation length on early and long-term urinary continence recovery after laparoscopic radical prostatectomy, several limitations should be acknowledged. The sample size was relatively small, which may have limited the ability to fully capture all factors influencing continence recovery. Although the follow-up period covered 24 months, it may be insufficient to evaluate the long-term effects of membranous urethra preservation on continence. The postoperative recovery of prostate cancer patients is a prolonged process, and continence function may undergo further changes over time due to factors such as scar tissue maturation and long-term neuromuscular repair. The inability of this study's follow-up period to observe these long-term changes may have resulted in an incomplete understanding of the relationship between preservation length and continence recovery. Future research could address these limitations by expanding the sample size to include more patients from diverse regions and with varied disease characteristics, enhancing the representativeness of results; extending the follow-up period to observe long-term effects; adopting a prospective study design to strictly control confounding factors and improve scientific rigor; and incorporating multicenter research to integrate resources and data from different institutions, further validating and refining the conclusions of this study.

## 6 Conclusion

In summary, this study clarifies that the preserved length of the membranous urethra during laparoscopic radical prostatectomy is closely associated with both early and long-term urinary continence recovery. Preserving a longer membranous urethra significantly promotes postoperative continence recovery, providing important theoretical and practical guidance for clinical surgery.

## Data Availability

The raw data supporting the conclusions of this article will be made available by the authors, without undue reservation.
